# Clinical application of ultrasound‐guided mediastinal lymph node biopsy through cervical mediastinoscopy

**DOI:** 10.1111/1759-7714.13717

**Published:** 2020-11-03

**Authors:** Yili Fu, Qingshan Chen, Zexing Yu, Honghong Dong, Xin Li, Qirui Chen, Bin Hu, Hui Li, Jinbai Miao

**Affiliations:** ^1^ Department of Thoracic Surgery Beijing Chao‐Yang Hospital, Capital Medical University Beijing China; ^2^ Department of Ultrasound Medicine, Beijing Chao‐Yang Hospital Capital Medical University Beijing China

**Keywords:** biopsy, lymph node, mediastinoscope, mediastinoscopic ultrasonography, ultrasound guidance

## Abstract

**Background:**

Cervical mediastinoscopy is useful for diagnosing lung and mediastinal disease. Ultrasound is a safe real‐time diagnostic tool widely employed in many surgical fields. Ultrasound was used in cervical mediastinoscopy in our cohort with satisfactory results. This study investigated the safety, feasibility, and availability of video‐assisted mediastinoscopy (VAM) combined with ultrasound for mediastinal lymph node biopsy.

**Methods:**

A total of 87 cases involving cervical mediastinal lymph node biopsy performed from November 2015 to May 2020, with complete clinical and pathological information, were retrospectively analyzed in the Department of Thoracic Surgery at Beijing Chaoyang Hospital. The cohort was divided into two groups: ultrasound‐guided biopsy under video‐assisted mediastinoscopy (UVAM) (44 cases) and routine VAM (43 cases). Operation time, biopsy number and nodal stations, postoperative complications, pathological conditions, and surgical difficulty were compared between the two nodal stations.

**Results:**

UVAM was significantly shorter and more lymph node specimens were obtained than with VAM. There was one case of fatal bleeding and two cases of right recurrent laryngeal nerve injury in the VAM group, and no postoperative complications in the UVAM group.

**Conclusions:**

When used with cervical VAM, ultrasound guidance assists physicians assess the space between lymph nodes, surrounding tissues, and large vessels systematically, making biopsy safer and easier, improving lymph node sampling, and decreasing postoperative complications. Furthermore, surgeons can easily learn and master this method.

**Key points:**

Significant findings of the study: Ultrasound was used in combination with cervical mediastinoscopy and the results showed that ultrasound guidance makes biopsy in patients safer and easier, improves lymph node sampling, and decreases postoperative complications.

What this study adds: Surgeons can easily learn and master this method.

## Graphical abstract

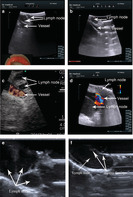

Ultrasound was used in combination with cervical mediastinoscopy and the results showed that ultrasound guidance makes biopsy in patients safer and easier, improves lymph node sampling, and decreases postoperative complications. Furthermore, surgeons can easily learn and master this method.

## Introduction

Video‐assisted cervical mediastinoscopy (VAM) is a technique used for diagnosing lung cancer and mediastinal diseases. This technique is commonly employed to identify the presence or pathological nature of mediastinal lymph node metastasis in patients with lung cancer whose chest CT indicates mediastinal lymph node enlargement. Endobronchial ultrasound‐guided transbronchial needle aspiration (EBUS‐TBNA) and endoscopic ultrasonography (EUS) are widely used in mediastinal lymph node biopsies. Mediastinoscopy is the gold standard procedure for diagnosing mediastinal lymph nodes in lung cancer^1^, ^2^, ^3^ and is essential for sampling enlarged nontumorous mediastinal lymph nodes in cases in which EBUS is not feasible, test results are negative, and further detection of diseased tissues and genetic testing are necessary.

However, mediastinoscopy does not easily distinguish lymph nodes from surrounding blood vessels and may increase the operative risk because of the narrow field of view and limited operating space. Although the incidence of bleeding using this procedure has been reported to be low,^4^, ^5^, ^6^ these limitations increase the risk of injury^7^ and the need for emergency thoracotomy or median sternotomy. Ultrasound is a safe and inexpensive diagnostic tool widely adopted in many surgical fields and can produce real‐time images without significant negative effects.^8^, ^9^ A major advantage of ultrasound is the ability to identify blood vessels.

Ultrasound is applied in combination with mediastinoscopy to better distinguish lymph nodes from surrounding blood vessels and reduce intraoperative injury^.^ ^10^ To further evaluate the safety, feasibility, and availability of this technique, we retrospectively analyzed data on surgical procedures conducted at our hospital. Furthermore, we determined that biopsy under ultrasound is safe and can determine the size and characteristics of tissues in real‐time.

## Methods

### Study design

From November 2015 to May 2020, 127 patients underwent mediastinal lymph node biopsy under conventional VAM or ultrasound‐guided VAM randomly in the Department of Thoracic Surgery, Beijing Chaoyang Hospital, of which 87 cases had complete clinical and pathological information. The number of male and female patients in our cohort was 33 and 54, respectively (sex ratio of 0.66), and the average age was 55.8 years (range: 26–78 years). The exclusion criteria was patients with a previous history of chest surgery. The inclusion criteria was patients with enlarged (≥1 cm) lymph nodes in the paramediastinum on contrast‐enhanced chest computed tomography (CT) or positive PET‐CT findings. Also, patients who did not receive a definite diagnosis using fiberoptic bronchoscopy, sputum examination, and cytological examination, or needed lymph node staging for advanced treatment. A total of 87 patients were divided into two groups: ultrasound‐guided VAM (UVAM) (44 cases) and conventional VAM (43 cases). This study was approved by the research ethics committee of Beijing Chaoyang Hospital. All patients signed their informed consent before enrollment into the study.

### Procedure

**Figure 1:**
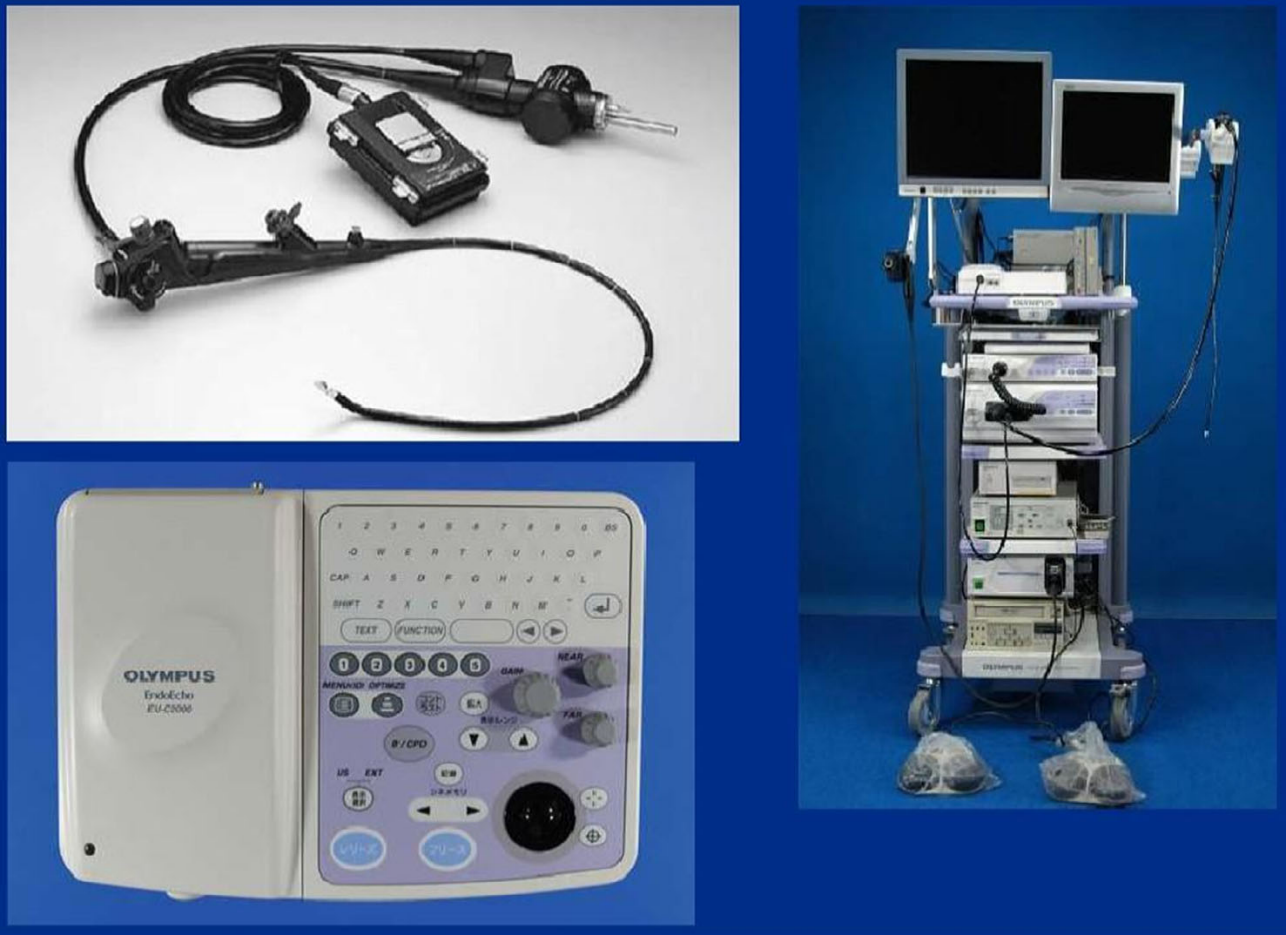
Ultrasonic probe preparation. Olympus endoscopic ultrasonography.

Cervical mediastinoscopy was performed using a single‐lumen endotracheal tube under general anesthesia. The ultrasonic device FF800 containing an 8666RF probe (BK Medical) and an EBUS probe (Olympus) (Fig 1) was sterilized at low temperature and used in all procedures. In the VAM group, after biopsy location based on CT images and surgical exploration (both sides of the trachea, under the carina, suspicious nodules, and lumps near the main bronchus), lymph nodes were detected and determined by puncture, and tissue specimens were biopsied. In the UVAM group, for surgical exploration, an ultrasonic device was used to identify the biopsy site and check local tissue thickness and surrounding tissues during punch biopsy, removing tissue as much as possible. Saline was injected around the ultrasound probe, so there was a certain space between the probe and the target tissue to ensure that the biopsy was real‐time under ultrasound monitoring. The surgical site was then washed with distilled water and iodophor solution to maintain hemostasis and sutured. After 24 hours monitoring, patients were discharged or underwent other procedures.^10^

### Data collection

Data on operation time, biopsy site, number of harvested lymph nodes, intraoperative and postoperative pathological results, complications, and the level of difficulty of the procedure assessed by surgeons and surgical assistants were obtained.

### Statistical analysis

Statistical analysis was performed by analysis of variance, *t*‐test (for continuous data), and chi‐squared test (for categorical data) using SPSS software version 22.0. A two‐sided *P*‐value of less than 0.05 was considered to indicate a statistically significant difference.

## Results

### Demographic data

There was no significant difference (*P* > 0.05) in age and gender ratio between the two groups (Table 1).

**Table 1:** Demographic and intraoperative data in our cohort

		VAM group	UVAM group	Student's *t*‐test/chi‐square test	*P*‐value
Age (years)		56.7 ± 11.4	54.9 ± 13.1	0.686	0.494
Gender	Male	15	17	0.132	0.717
	Female	28	27		
Operation time	Minutes	31.5 ± 10.7	25.7 ± 6.2	3.144	0.002
Groups of lymph nodes	Mean	2.4 ± 0.9	3.3 ± 1.1	4.598	<0.001
Number of lymph nodes	5^†^	1	6	20.214	<0.001
	4^†^	3	14		
	3^†^	12	15		
	2^†^	23	7		
	1^†^	4	2		
Nodes harvested during biopsy		2.9 ± 1.1	4.7 ± 1.6	6.177	<0.001
Average time necessary to harvest each lymph node	Minutes	11.6 ± 3.0	5.92 ± 1.7	10.708	<0.001

^†^ Number of different stations to obtain lymph nodes.

### Intraoperative data

**Figure 2:**
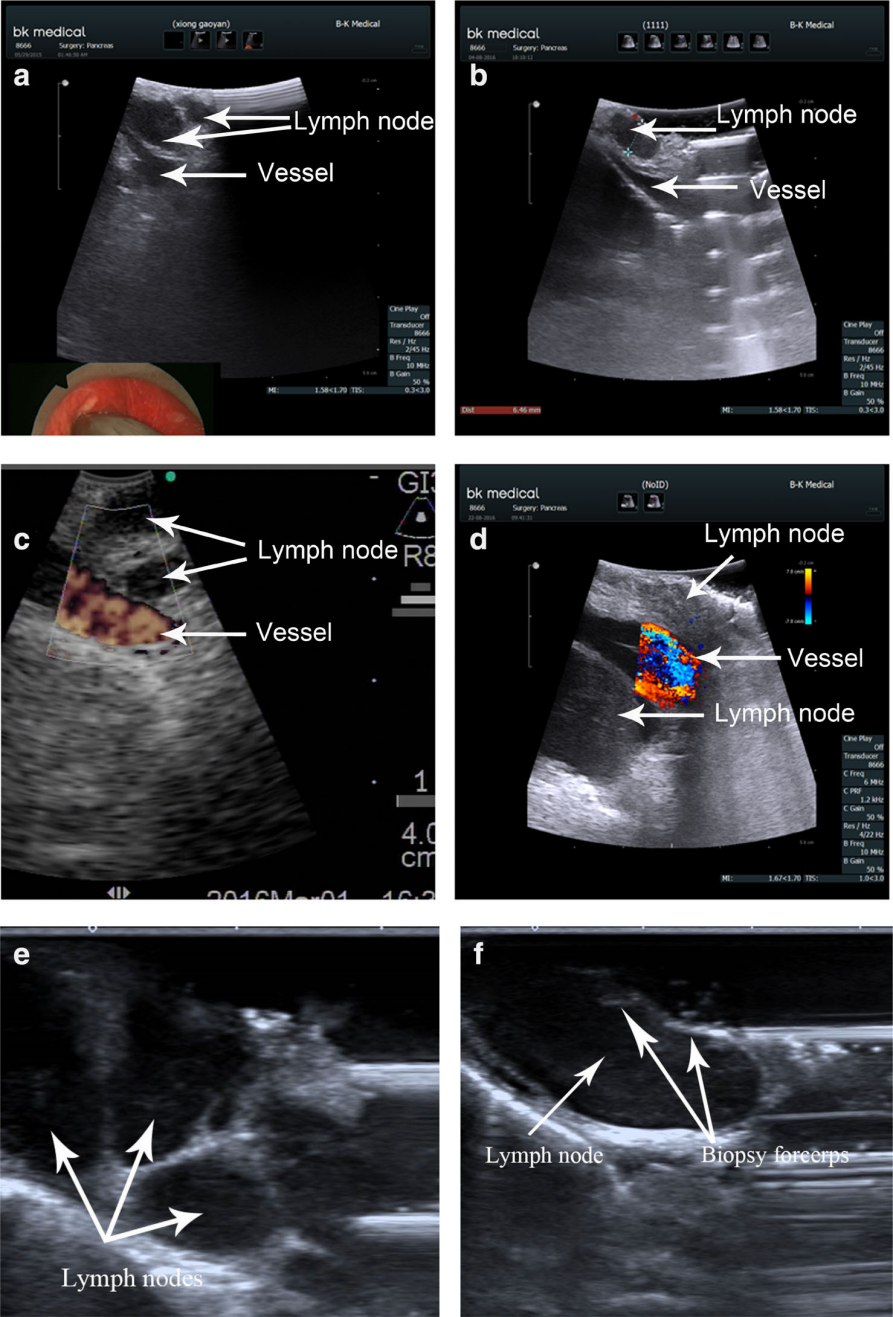
Ultrasonic measurement of mediastinal lymph nodes. (**a**, **b**) 2R lymph nodes behind a vessel. (**c**, **d**) Ultrasound assessment of the blood flow of lymph nodes, with clear distinction between lymph nodes and surrounding blood vessels. (**e**) Multiple lymph nodes visualized on ultrasound. (**f**) Biopsy forceps visualized on ultrasound.

In the UVAM group, the number and size of lymph nodes and the relationship with large blood vessels were assessed by ultrasound (Fig 2). The operation time was significantly shorter in the UVAM group (25.7 ± 6.2 minutes vs. 31.5 ± 10.7 minutes, *P* < 0.001). No. 2 (bilateral), No. 4 (bilateral), and No. 7 lymph nodes were detected in both groups. However, most patients (35/44, 79.5%) in the UVAM group had more than three groups of lymph nodes, whereas most patients in the VAM group (16/43, 37.2%) had two groups of lymph nodes, and the difference was statistically significant (*P* < 0.001). In addition, the number of harvested lymph nodes was significantly higher in the UVAM group (Table 1).

### Assessment of surgical difficulty

The number of surgeons who rated the level of difficulty of the surgical procedure as low was significantly higher in the UVAM group (27/44 [61.4%] vs. 2/43 [0.05%]; X^2^ = 42.284, *P* < 0.001). The number of surgical assistants who rated the level of difficulty as moderate or high was significantly lower in the UVAM group (13/44 [29.5%] vs. 43/43 [100%]) (Table 2).

**Table 2:** Assessment of surgical difficulty

		VAM group	UVAM group	Chi‐square test	*P*‐value
Level of difficulty of the operation assessed by surgeons	High	20	0	42.284	<0.001
	Moderate	21	10		
	Low	2	27		
Level of difficulty of the operation assessed by surgical assistants	High	31	3	35.599	<0.001
	Moderate	12	10		
	Low	0	31		

### Postoperative data

The rate of pathological false‐negative results and postoperative complications was nonsignificantly higher in the UVAM group (*P* > 0.05). In the VAM group, there was one case of massive hemorrhage with an enlarged lymph node found to be adhered to the brachiocephalic artery during the operation which required emergency median sternotomy, and two cases of right recurrent laryngeal nerve injury after surgery, with improvement after three months. In the latter two cases, bilateral superior mediastinal lymph nodes were enlarged during surgery, and biopsies were performed on both sides. Postoperative laryngoscope confirmed that the right recurrent laryngeal nerve was damaged. There were no complications in the UVAM group. All patients with pathologically negative lymph nodes underwent lobectomy and systematic mediastinal lymphadenectomy, including five patients in the UVAM group and seven patients in the VAM group. The results of pathological examination postoperatively showed that three patients (7%) in the VAM group had positive lymph nodes (one in station 4R and two in station 7). There were no false‐negative cases in the UVAM group (Table 3).

**Table 3:** Postoperative pathology

Pathology	VAM	UVAM	Chi‐square test	*P*‐value
Squamous cell carcinoma	5	4	1.689	0.890
Adenocarcinoma	14	16		
Small‐cell lung cancer	4	6		
Sarcoidosis	8	10		
Lymphoma	5	3		
Negative (false negative)	7 (3)	5 (0)		
Pathology diagnostic accuracy	93.2%	100%		

## Discussion

VAM has great clinical value for mediastinal lymph node and tissue biopsies and is commonly used to diagnose lung diseases.^1^ However, general thoracic surgeons who lack knowledge of mediastinal anatomy and practical experience have difficulty using this technique because the large number of blood vessels and limited operative space in the mediastinum can increase surgical risk. Furthermore, VAM biopsy may cause large blood vessel injury, especially in the brachiocephalic trunk artery, anterior vena cava, and pulmonary artery. Although the incidence rate has previously been reported to be 0.6%–3.0%, patients with these complications need emergency thoracotomy or median sternotomy.^2^

Intracavitary ultrasound is advancing rapidly and is widely applied in surgical operations to distinguish blood vessels from solid tissues.^11^, ^12^ Previous studies have shown that ultrasound combined with mediastinoscopy is useful to determine the T stage of lung cancer and assess the extent of tumor invasion in surrounding tissues^.^ ^13^ On this basis, we used physiological saline as medium to obtain tissue biopsies under real‐time ultrasound guidance. This study determined the relationship between lymph nodes, peripheral nerves and blood vessels to improve the operability and safety of surgery. We found that ultrasound combined with VAM significantly reduced surgical and biopsy time for average lymph nodes compared with conventional VAM. A possible reason is that less mediastinal tissue is dissected during surgery. Ultrasound combined with CT and surgical exploration can improve the visualization of lymph nodes in the mediastinum and increase diagnostic accuracy. Although the ultrasonic probe required space to move in the mediastinum because of its size, the anterior aspect of the trachea needed to be separated; however, it was not necessary to expose most of the lymph node. Real‐time ultrasonographic guidance increases the safety and accuracy of mediastinal lymph node biopsy and reduces the risk of iatrogenic injury.

UVAM increased samples of biopsy tissue, and the number of lymph nodes sampled was significantly higher using UVAM than VAM. A possible reason is that ultrasound improved the visualization and biopsy of Nos. 2, 4, and 7 lymph nodes, as well as smaller lymph nodes, which are usually poorly visualized using VAM. Although ESTS guidelines for preoperative lymph node staging of non‐small cell lung cancer recommend exploring nodal stations 2R, 2L, 4R, 4L, and 7 to detect metastases,^14^, ^15^, ^16^ it is difficult to achieve totally and is usually simplified in clinical practice for two reasons. First, positive lymph nodes in stations 2R/2L, 4R/4L, and 7 are N2/N3 for unilateral or bilateral nodes. Therefore, if one group is confirmed as positive during frozen‐section examination, further sample collection is unnecessary. Second, mediastinal lymph nodes are usually embedded with nerves and large blood vessels, so that operators may have difficulty distinguishing small lymph nodes from tortuous vessels. In contrast, ultrasound improves the visualization of smaller lymph nodes and the distinction between lymph nodes and surrounding vessels, which improves operator confidence and surgical success. There were three (7%) false‐negative cases in the VAM group, which is in agreement with a previous study, wherein the incidence of false‐negative cases using mediastinoscopy was 6.8%,^17^ with no false‐negative cases in the UVAM group. The absence of a significant difference between these groups may be due to the small sample size.

Even experienced thoracic surgeons may feel apprehensive if a lymph node is not confirmed during VAM. Puncture is effective but has limitations. First, a small puncture may cause large‐vessel bleeding. Although compression hemostasis is effective, the operation time is long, and may affect a surgeon's confidence. Second, in light of the limited space of the mediastinoscope, operators cannot easily control the angle and depth of the probe needle during a biopsy, which decreases puncture accuracy. One patient in the VAM group was punctured three times at different sites without bleeding; nonetheless, massive hemorrhage occurred during protractor biopsy. The surgeons adopted gauze packing and median sternotomy immediately after surgery and found that enlarged lymph nodes that adhered to the posterosuperior brachiocephalic artery were slightly deformed. Part of the vessel wall was removed during biopsy. We believe that puncture at different sites without bleeding indicates the absence of blood vessels around the lymph nodes. This situation can be avoided using ultrasound because this technique allows accurate determination of lymph node size. Combined with the Doppler effect, surgeons can more accurately measure the lymph node size, and the distance between lymph nodes and large blood vessels. Compared with conventional VAM, operator confidence is improved using ultrasound and assistants can easily learn and master this technique.

Our study has some limitations. First, it was retrospective and therefore some points of view may be biased. Second, the ultrasound probe was not specific for the mediastinoscope and for this reason there were some blind spots. Third, surgical difficulty was assessed subjectively. In the UVAM group, there was no specialized ultrasonic device for mediastinoscopy. and therefore an abdominal ultrasound probe and an EBUS probe were used.These probes were used in the limited space of the mediastinoscope and their performance was similar regarding inspection scope and differentiation between lymph nodes and surrounding tissues. Notwithstanding this, we determined that because the EBUS probe was smaller it was consequently better for assessing No. 7 lymph nodes, whereas the abdominal ultrasound probe was more rigid and better for assessing No. 2 lymph nodes.

Ultrasonic techniques allow surgeons to detect the relationship between lymph nodes and surrounding large blood vessels during biopsy, improving the safety and simplicity of the operation, increasing the number of harvested lymph nodes, and reducing the risk of intraoperative injury. The results of this research prove the safety, feasibility, and wide availability of ultrasound guidance for mediastinal lymph node biopsy.

## Disclosure

The authors declare that there are no conflicts of interest.
